# Factors That Impact Measures of Grit among Nursing Students: A Journey Emblematic of the Koi Fish

**DOI:** 10.3390/ejihpe10020041

**Published:** 2020-04-05

**Authors:** Daniel Terry, Blake Peck

**Affiliations:** School of Nursing and Healthcare professions, Federation University Australia, 3350 Ballarat, Australia; b.peck@federation.edu.au

**Keywords:** grit, nursing, students, clinical, academic, performance

## Abstract

Grit is the capacity to persevere, to have passion, and be committed to achieve goals long-term regardless of adversity or challenge. Grit provides an insight into why some nursing students succeed academically or clinically, while others do not. This quantitative cross-sectional correlational study measured levels of grit among nursing students undertaking a three-year bachelor’s degree program. All students (n = 2349) within the program were invited to complete a questionnaire which included the short grit scale (Grit-S) which measured each student’s level of perseverance and passion. Overall, it was highlighted that increased levels of grit correlated with an increase in the student’s year of study, greater perceived clinical and academic performance, not using television as a motivator for entry to nursing, being lower on the socio-economic spectrum, and being older in age. Grit was found to develop exponentially as students entered second and third years, suggesting that a balance of constant academic and clinical challenge was an impetus for many to achieve in the face of adversity, and is reminiscent of the journey of the koi fish. This paper culminates in a call for educators to consider the inclusion of creative grit forming challenges that focus on developing a student’s sense of open-mindedness within first year of undergraduate nursing programs.

## 1. Introduction

Grit is the capacity to persevere, to have passion, and be committed to achieve goals long-term regardless of adversity or challenge [1], however Robinson [2] more clearly describes grit as the consistency of interest and perseverance of effort. Grit is considered more than resilience, which is defined as an optimism to continue, even when times are tough, which leads to success. Instead grit is an intuitive trait encompassed by a determination that individuals use to confront challenging tasks not just for the short-term, but for an extended period of time. It is the ability to continuously forge ahead irrespective of the challenge or difficulties which may lay in front of a person, and is symbolic of the koi fish, who despite the challenges they encounter, possess a persistence in swimming against streams and climbing rushing waterfalls [1,3].

Grit is more than stubbornness or strong-mindedness to achieve, regardless of the expenditure of time or energy; it is about realising and accomplishing key longer-term pursuits, and still possessing the flexibility to meet those short-term day-to-day goals [1,3]. Grittier people have a tendency, when encountering setbacks, poor feedback, disappointments, or plateaus in their pursuits, to seek understanding in order to continually move forward to improve—they do not deviate from their goals [1,3]. 

Duckworth and Peterson [3] first introduced the construct of grit and showed that it predicted achievement more than an individual’s talent alone. Furthermore it was shown that grit highly correlates with other traits such as perseverance, consistency, and the big five personality traits of conscientiousness [3,4]. It has been suggested that grit is similar to conscientiousness or that grit is or mimics conscientiousness [5], however, it is further argued that grit, although within the “conscientiousness family”, is theoretically different in that it is the passion or tenacity to achieve long-term goals, rather than simply a personality trait [6,7,8]. In addition to this, grit has been argued to be more likely to determine success beyond conscientiousness, intelligence, and physical abilities [3,9]. This has been demonstrated through a number of studies that have highlighted higher levels of grit, rather than other factors or traits as being predictive of achievement such as better spelling bee performance, higher grade point averages, higher levels of high school graduates, less hours watching television among adolescents, military cadet retention, and longer term employment [3,4,9,10,11]. 

Developing or increasing grit remains complex and often elusive as it is not a homogeneous process, however, a number of principles have been distilled that help to understand how it may be cultivated, which includes educational contexts. It requires a change and growth in mindset [1,5], with factors having the most impact being greater self-belief, clear goal setting, improved social connectedness and self-regulation or ability to control one’s behaviours, emotions, and thoughts. To achieve these factors, one must set achievable smaller, medium and longer-term goals, while using reflection to ascertain accomplishment and future action [1,3]. Further, one must use language that fosters resilience and success, be around and seek help from positive people, where positive thinking is the norm, and nourish flexible thinking. When a growth in mindset occurs, particularly in education, individuals begin to perceive a difficulty as a learning opportunity instead of a challenge to overcome, individuals respond with constructive thoughts and behaviour, showing persistence rather than being defeated [1,5,12]. 

It has been suggested that moderate challenges are often required to motivate gritty people—if the task is perceived as “too easy”, ill-defined, unusual, or even overly difficult in terms of expenditure of effort for relative output, then the reward is null and void [1,5]. Gritty people will work harder when an incentive of a challenge exists. Perhaps contrary to popular thought, individuals do not necessarily require the experience of trauma, such as poverty, discrimination, or tragedy, for grit to be more fully enacted or developed, rather, encountering “appropriate” levels of challenge or difficulties, with adequate and consistent support, allows individuals to grow and develop grit [1,5]. While grit can be developed from exposure to trauma and very difficult challenges, it also requires a reciprocal level of encouragement and respectful support to be fully enacted [1,3]. 

Grit, although examined widely and taught within the US, remains a new construct in both the nursing profession and Australia. The global demand for nurses is projected to experience a significant shortfall by 2030, particularly in westernised countries where the population is rapidly ageing [13,14]. These shortages are made worse with up to 25% of new nurses leaving the profession within five years of graduation [13,14]. Nurses, although shown to develop grit through their professional practice [15], may still leave due to workload, workplace culture, and emotional strain. Thus, there is a need for better insights into how to more adequately prepare students for the rigors of the nursing profession in the higher education setting. This should begin with a sense of how gritty nursing students really are on the whole, as well as those factors that impact grit among undergraduate nursing students [16].

Presently there exists very little research literature that purports to articulate the general levels of grit and those factors that may impact the grittiness of nursing students. The review conducted by Stoffel and Cain [16] identified 11 journal articles that were focussed on resilience and hardiness, however only one commentary article [15] specifically discussed and one [2] researched the impact of grit among nursing students. Although grit has been explored among general university populations and among military cadets where it was found to have a relatively positive impact in the US [1,2,3,4,5,17], there has been scant insight among nursing students, particularly in Australia. As such, the aim of this research was to investigate the overall sense of the levels of grit among a cohort of nursing students in higher education, as well as those factors that correlate with higher levels of grit or grittier nursing students.

## 2. Materials and Methods 

A quantitative cross-sectional correlational study examined grit among students undertaking a Bachelor of Nursing (three-year) program at an Australian university with campuses in rural, regional and peri-urban centres.

### 2.1. Sample 

The whole cohort of nursing students (n = 2349) were asked to participate in the study by completing an online questionnaire examining grit. The nursing student cohort consisted of 18.4% (n = 422) rural and regional students, 86.1% (n = 2023) female students, 0.9% (n = 22) indigenous students, 12.3% (n = 291) international students, including 21.8% (n = 514) students entering the program through direct pathways from high school. For the study to demonstrate it had sufficient power to detect a 5% absolute difference within and between groups the sample size needed was n = 300, alpha (2 tailed) = 0.05, margin of error = ±5%.

### 2.2. Data Collection Instrument

A questionnaire was used to collect the data, which encompassed key demographic questions, inclusive of year of program, place of residence, gender, year of birth, marital status, employment status, income, where the students saw themselves working in the future as nurses, and if they had a healthcare card. A healthcare card is an Australian government scheme which entitles Australian citizens who are low-income earners or who are eligible through other means, such as having child or carer obligations. Eligibility is based on an assessment which includes key parameters such as income, age, and/or individual or family circumstances. Recipients are entitled to concessions for healthcare, pharmaceutical cost, and additional concessions on essential services such as electricity, public transport, and some taxes. The healthcare card is often used as an alternative measure or indicator of low income or socio-economic status [18,19].

In addition to key demographics, the questionnaire included a number of scales. These included the general self-efficacy scale (GSE-10) which contains 10 general self-efficacy questions which were used to provide insight into the overall self-efficacy of the students (reliability α = 0.888) [20]; the internal external locus of control-4 (IE-4) scale which measures the psychological concept regarding how strongly nursing students believe they have control over the situations and experiences that affect their lives (reliability α = 0.304) [21]; and the big five inventory extra-short form (BFI-2X), where participants needed to self-rate themselves against 15 key statements and how true the participants felt the statements were about themselves. These statements related to the five personality traits of extraversion, agreeableness, conscientiousness, neuroticism, and open-mindedness. Items are measured on a five-point scale with categories ranging from agree strongly to disagree strongly [22]. Overall, the reliability of the personality traits had good acceptability with coefficients for extraversion = 0.453, agreeableness = 0.583, conscientiousness = 0.584, neuroticism = 0.620, and open-mindedness = 0.312. The reliability of the subscale of extraversion and open-mindedness were lower than anticipated (range 0.510–0.720) [22], however it is noted that scales with a small number of items have a propensity to reach adequate reliability [23]. The last key measure was the eight-item short grit scale (Grit-S). The scale was initially developed by Duckworth and Quinn (2007) to measure trait-level perseverance and passion (reliability α = 0.755) [3]. Overall, the questionnaire was designed to be completed between 15–25 min. 

### 2.3. Data Collection

Data were collected between June to August 2019. Administration staff were provided with an invitation letter from the researchers to forward to all nursing students via email. The aim was to ensure there was no coercion from researchers toward students. The invitation included a web-link to the information regarding student participation, where students gave informed consent, and could then undertake the questionnaire. A series of follow-up emails were sent via administration staff to nursing students each week for five weeks until an adequate sample size (n ≥ 330) was obtained to meet 95% CI (MOE ± 5%). If students did not complete the questionnaire, this data were excluded.

### 2.4. Ethical Considerations

Ethical approval was gained through the Federation University Australia Human Research Ethics Committee (Approval #18-017). The invitation to participate in the anonymous questionnaire was sent at the commencement of the mid-year break to reduce bias or impact on students’ studies and reduce the risk of coercion. Participants were not offered incentives to participate. 

### 2.5. Data Analysis

Data were analysed using statistical package for the social sciences (SPSS, Version 22.0). Statistical tests such as Cronbach’s alpha (α) were used to test scale item reliability. Furthermore, an independent sample t-test, and one-way ANOVAs were used to analyse data and examine group differences. Lastly, multiple regression was used to observe the association between grit and a number of predictor variables. Preliminary analysis was undertaken to ensure no violations of assumptions were present. Significance was agreed to be at *p* ≤ 0.05 (two-tailed).

## 3. Results

A total of n = 2349 students undertaking a bachelor of nursing degree were asked to take part in the school-wide questionnaire with n = 544 responding, which yielded a response rate of 23.2%. After incomplete questionnaires were excluded the remaining n = 435 partially or fully completed questionnaires led to a response rate of 18.5%. The demographics of the participants are highlighted in Table 1 and demonstrate that over half (n = 265) of the participants were between 20 and 39 years of age, with under a third (n = 105) of participants born overseas. More than a third indicated they were single (n = 123), while more than half (n = 221) were working either in a full time, part time, or casual capacity. 

**Figure 1:**
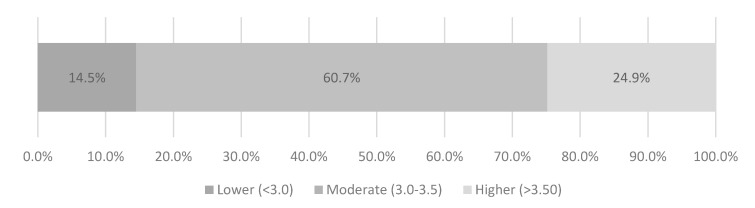
Mean levels of grit among students.

The outcome of the Grit-S highlighted mean grit score was 3.8 (range 2.5–5.0) and among the respondents, 24.9% demonstrated higher levels of grit (>3.5), followed by 60.7% who had moderate levels of grit (3.0–3.5), while 14.5% had lower levels of grit (<3.0), as outlined in Figure 1.

**Figure 2:**
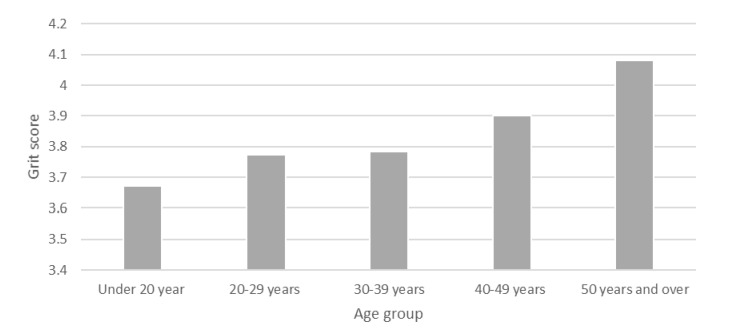
Mean levels of grit according to student age groups.

The examination of grit levels between various groups including sex, age group, marital status, first family member to attend university, status of employment, being overseas born, and being an enrolled nurse revealed there were no significant differences in mean scores of grit for many demographic factors. Although there was no statistical significance identified for mean levels of grit indicated between age groups, it was shown that as student age groups increased, so did their levels of grit (Figure 2).

Further examination highlighted that there were significant differences in the levels of grit according to the year level of students, where first year students’ mean level of grit was significantly lower than both second and third year students. Similarly, levels of grit were significantly different between self-rated performance (academic and clinical), where students who rated their academic and clinical performance as average had lower scores than those who rated it much higher. Lastly, students who indicated that television was a motivator for wanting to become a nurse had significantly lower grit levels than those who were not motivated by television to enter nursing as a profession (Table 2).

Multiple regression analysis underscored several significant predictor variables of grit and indicated that they explained 49.7% of the variance that can be attributed to factors important to grit F(7,227) = 31.690, *p* = 0.000. As outlined in Table 3, having a healthcare (low income) card, agreeableness, conscientiousness, general self efficacy, and locus of control were significant predictors of grit among the nursing students. This would imply that students with high levels of grit are 1.300 times more likely to be conscientious, be 1.221 times more likely to be agreeable, 1.03 times more likely to have greater levels of general self-efficacy, and 1.155 times more likely to have internal locus of control than those with lower levels of grit. Interestingly, those students that are 1.13 times more likely to possess a healthcare card had higher levels of grit.

## 4. Discussion

Nursing students in this study showed a collective moderate to high level of grit. This high level of grit is perhaps unsurprising, given that nursing students have consistently showed high levels of conscientiousness, which is often considered in terms of being cautious, methodical, well planned, and responsible. In previous studies, students with high levels of conscientiousness sought outcomes that are achievable, were found to be more careful in decision-making, and less impulsive [24]. The findings suggest there are some unremarkable, yet interesting, areas for deeper consideration. 

It is noted that although not significant, there was a correlation between increases in grit with increasing age of students, which further confirms previous work that suggests grit increases with life experience. Where longer-term goals have been made and met, older students are able to draw from these experiences in order to answer the grit scale items and to therefore demonstrate greater levels of grit when compared with their younger counterparts in the nursing program, which may be beneficial when entering practice [4,17,25,26]. Of interest, one Australian study [27], found that students who were first in their family to attend university showed increased levels of the factor “effort”, which is central to grit. However, this was not the case in this study, which was aimed at a nursing student cohort at a single university site, while the study by Hodge et al., (2018) was Australia-wide but did not indicate a specific course that the students examined were studying.

Furthermore, it was also noted that second and third year students had significantly higher levels of grit than their first year student colleagues. It has been observed that grit is more greatly developed where scholarship and learning are both demanding of the student, yet also supported in achieving success [1,3,4]. It was noted that levels of grit between second and third year students were relatively similar, therefore what is posited is that among nursing students, the second year of the program represents perhaps the time within the nursing program where grit may undergo its greatest development. This would seem consistent with other work by Admi, Moshe-Eilon [28], who suggested that the second year of the program is typically the most challenging year of a nursing curriculum. In terms of grit, therefore the second year of the program might represent a balance of incentive and challenge that harnesses the grittier elements of the collective student cohort. Duckworth [1], when speaking of parenting, suggests that it is “tough love” where grit is both forged within the crucible of demand by a parent on a child. Likewise, in higher education, particularly in a student’s second year, greater demands are placed on students by the university or academics to meet and achieve goals. However, grit is also about a child being nurtured through care and support by a parent; similarly university structures and academics provide safe spaces where students can practice, develop, falter and learn [26]. 

It is this gritty perseverance that is developed from a growth mindset—the development of skill or talent through deliberate practice and hard work, where obstacles are perceived as challenges that can be overcome rather than impassable, which has been shown to improve academic success [26,29]. These findings are confirmed within the present study, where grit scores were highest among students who—arguably portraying an open-mindset—perceived their academic and clinical performance to be more advanced when compared to those who perceived their performance as average. This is observed in a number of other studies where high levels of grit were related to greater levels of course engagement and more advanced clinical skills among those studying nursing [5,16,30,31]. 

Grit levels were also noted to be higher among students who were not motivated by television to enter nursing as a profession. Previous research [17,32] has suggested grit is negatively associated with hours watching television, and Lee and Sohn [29] postulate that it is the act of watching, which inhibits study quality, that leads to poorer academic performance [16]. This study is the first to suggest television, specifically medical television, as a motivating factor for undertaking nursing as a career has an impact on levels of grit. However, it may be concluded that students who were motivated by television, may in fact engage in a higher number of hours watching television than those who were not motivated by television to enter nursing. This finding may therefore further confirm the negative relationship between television and grit. Regardless of the number of hours spent watching television—irrespective of the content—what is uncovered is that those students who indicate they are motivated by television to enter the nursing profession are more likely to have significantly lower levels of grit than their non-watching counterparts. 

Overall, the findings suggest that students who have higher levels of agreeableness, conscientiousness, self-efficacy, and have an internal locus of control have a propensity to have higher levels of grit. It is noted that conscientiousness as a personality trait is a vital contributing facet of grit [9,10,17,26,29] and academic performance among higher education students [10,25,33]. In addition, the findings remain informative in understanding that locus of control, the capacity for initiative and motivation, and persistent performance [34], remains an essential facet of grit [17,34]. Furthermore, both agreeableness and self-efficacy have been shown to correlate with grit and together have an impact on overall higher education student performance [9,30].

It was noted that students who were more likely to possess a healthcare card express higher levels of grit across the cohort. It has been demonstrated by Anderson, Turner [34], that students from low income backgrounds have diverse levels of locus of control and “fate control”—an individual’s sense of control over their own environment. In this same vein, grit levels within this cohort of students was observed to be heterogeneous among those who are eligible for and possess a healthcare card. It is vital to note that previous studies were focused on low income high school students, however, what is elucidated from the literature is that success is achievable regardless of disadvantage with increases in grit over time and as goals are achieved and successes experienced [35]. As such, if a student is in possession of a healthcare card the present study would suggest that higher levels of grit are likely to be attained across the education journey for this cohort of students. It may also be surmised that students who possess a healthcare card and choose to attend university may already have higher levels of grit, or they have the capacity to develop greater levels of grit as they enter higher education where the drivers of grit, demand and support, are enacted [36]. 

### Limitations

The university where the study was conducted is situated across rural, regional and peri-urban locations. As such, the student cohort may have a higher percentage of students who are from rural settings. This may have an impact on the data in terms of the generalisability of the findings, specifically to other universities which are situated in more urban centres. Although adequate numbers of students responded, the response rate may impact the findings being representative of the whole student cohort. To increase response rate without increasing coercion among students who are studying, the questionnaire may potentially be administered outside any intense study period, however this may also have limitations.

## 5. Conclusions

Considered a non-cognitive drive that keeps people committed to a long-term task in the face of difficulty, grit has been shown elsewhere to be an insightful measure of both potential and actual success, particularly in terms of an individual’s longevity or “stick-ability” in a profession. Interestingly, grit is recognised as a factor amenable to change that can be harnessed and developed among individuals in an effort to ensure success over time. Despite this, and in the context of an ever-worsening global nursing shortage, studies of grit in nursing remain scant. This study sought to identify the overall levels of grit among a cohort of nursing students and to investigate what factors impact the levels of grit or grittiness among nursing students. The findings suggest a significant difference in levels of grit with increases consistent with the year of study, the degree to which an individual rated their academic and clinical performance, not using television as a motivator to enter the nursing profession, and having a lower socio-economic status. 

A closer examination of key personality and demographic variables suggests agreeableness, conscientiousness, general self-efficacy and locus of control all correlate positively with increased levels of grit. While studies have identified that conscientiousness and agreeableness are much less amenable to change, general self-efficacy and locus of control have been shown to be open to some degree of change. Similarly, the current study suggests that grit is also open to development. Measures of grit increased among students across the three years of the program with a significant rise identified between the first and second years. We postulate that this sharp rise represents a fertile period of development of grit on the basis of providing enough of a challenge to stimulate grit, while at the same time providing sufficient support among those that are prepared to accept the challenge.

This process is emblematic of the journey taken by the koi fish. Symbolic of many things in Asian culture, the koi are known for their tenacity in being able to climb rushing streams and waterfalls. They are said to have swum against the current of the Yellow River in China, progressively gaining strength as they persevered against the force. When they reached a waterfall at the end of the river many of the koi turned back. The remaining koi refused to give up attempting to reach the top of the waterfall. The mythology suggests that after one hundred years of jumping only one koi made it to the top and was turned into a golden dragon, the image of power and strength. 

Here the similarities of the development of grit and the koi fish are palpable. Students swimming against the current of academic and clinical workloads, while at the same time juggling expectations outside of university life creates the necessary challenge that makes the students stronger. Certainly the findings identified here would suggest that the metaphorical waterfall exists between first and second years of the program, where the students tended to identify the greatest change in their levels of grit, perhaps as a function of the increasing current of work that is typical of second and third years of study in bachelor of nursing programs. 

This study suggests that grit offers a rich stream for consideration in building a nursing workforce. Grit is a characteristic that is open to development and correlates strongly with other well-trodden measures of student success and professional longevity. What is needed are studies that provide some robust evidence for the impact of various interventions to increase grit among students, such as developing an open-mindset by way of establishing short-term goals that provide opportunities for detailed feedback and reflection, as well as targeting the practice of nursing skills, to name a few. The findings from this study would suggest that the cohort studied gains grit by actively facing an increasing current against which students in the first year of the program must work in order to see their metamorphosis to a golden dragon come to fruition. 

## Figures and Tables

**Table 1:** Student demographics.

Demographic Information	Frequency	Percentage (%)
Year of program (n = 434)		
1st year	149	34.3
2nd year	164	37.8
3rd year	121	27.9
Gender (n = 362)		
Female	329	90.8
Male	31	8.6
Other	2	0.6
Age (years) (n = 385)		
Under 20 years	32	8.3
20–30 years	147	38.2
30–39 years	118	30.6
40–49 years	61	15.8
50 years and over	27	7.0
Born in overseas (n = 362)		
No	257	70.9
Yes	105	29.1
Speak English as a second language (n = 200)	70	16.1
Marital status (n = 346)		
Single	123	35.5
Married/Partnered	200	57.8
Divorced/Separated	19	5.5
Other	4	1.2
Highest level of education (n = 349)		
Secondary school (year 12 or less)	108	30.9
Vocational education or trade training	198	56.7
Bachelor degree or above	39	11.1
Other	6	1.7
First in family to attend university (n = 360)	187	51.9
Employment status (n = 399)		
Not in paid labour force	48	12.1
Casual employee (no guaranteed hours of work)	130	32.6
Part-time employee (less than 38 h week)	167	41.9
Full-time employee (38 h a week)	54	13.5
Currently an enrolled (Division 2) nurse (n = 356)	67	18.8
Current after tax income (AUD$) a week (n = 376)		
Less than $400	142	37.8
$400–$799	165	43.9
$800–$1499	62	16.5
More than $1500	7	1.9
Healthcare (low income) card (n = 360)	137	35.8
Television a motivator to enter the profession (n = 426)	96	22.5

**Table 2:** Comparison of mean grit scores between groups.

Factor	Group 1	Mean Grit Score	Group 2	Mean Grit Score	Test (df) Statistic	*p* *
Year of program	1st Year	3.69	2nd Year	3.86	F(2,335) = 4.035	*0.050*
	1st Year	3.69	3rd Year	3.89		*0.032*
Perceived performance						
- Academic	Average	3.59	High	3.91	F(2,325) = 10.670	*0.000*
- Clinical	Average	3.50	High	3.91	F(2,309) = 14.425	*0.000*
Television a motivator	Yes	3.61	No	3.85	t(305) = 2.563	*0.011*

* *p* < 0.05.

**Table 3:** Factors that impact grittiness of nursing students.

Predictor	OR	95% CI	*p*-Value
Healthcare (low income) card	1.136	1.013–1.275	0.029 *
Extraversion	0.993	0.921–1.070	0.852
Agreeableness	1.221	1.119–1.333	0.000 *
Conscientiousness	1.300	1.195–1.423	0.000 *
Neuroticism	0.953	0.887–1.024	0.189
Openness	0.968	0.887–1.056	0.462
General Self Efficacy	1.032	1.016–1.048	0.000 *
Locus of Control	1.155	1.038–1.286	0.009 *

* *p* < 0.05.
